# Marine Natural Products as Anticancer Agents 3.0

**DOI:** 10.3390/md23060243

**Published:** 2025-06-05

**Authors:** Celso Alves, Marc Diederich

**Affiliations:** 1 MARE—Marine and Environmental Sciences Centre/ARNET-Aquatic Research Network, ESTM, Polytechnic University of Leiria, 2520-630 Peniche, Portugal; 2 Department of Pharmacy, College of Pharmacy, Seoul National University, 1 Gwanak-ro, Gwanak-gu, Seoul 08826, Republic of Korea

**Keywords:** cytotoxicity, semi-synthesis, apoptosis, immunotherapy, in vivo, mitochondrial dysfunction, motility, co-administration, anticancer

Cancer represents a major global health challenge, with 20 million new cases and 9.7 million deaths reported in 2022. By 2050, the number of new cancer cases will exceed 35 million [1]. Cancer is commonly understood as a single disease, but it comprises over 100 distinct pathological conditions, each with unique characteristics. This complexity makes it difficult to develop universal, effective treatments and classify cancer as a uniformly treatable condition. The therapeutic strategy for each type of cancer may differ depending on the nature and stage of the disease and often requires the combination of multiple therapeutic approaches, including radiotherapy, chemotherapy, hormone therapy, anti-angiogenic therapy, immunotherapy, and stem cell transplantation [2]. Consequently, the development of precise, individualized treatments and increased investment in cancer drug research are essential to fight this burden and reduce global cancer mortality. Despite the availability of large libraries of synthesized compounds, they have not been as successful in producing new drugs as many had hoped. In contrast, natural products, including those of marine origin, continue to play a key role in the discovery and development of novel anticancer medicines. They offer several advantages in drug discovery and development, such as remarkable structural diversity, the potential for multi-target effects, the ability to overcome drug resistance, and the specificity to interact with key targets associated with carcinogenesis [3].

The Special Issue “Marine Natural Products as Anticancer Agents 3.0” comprises eleven publications, three reviews, and eight original research articles, focusing on the anticancer activities of marine natural or derived compounds. The original research articles in this collection study the effects of these substances in silico, in both 2D and 3D in vitro cellular and in vivo models, across a range of solid and non-solid tumors. Forty-one marine natural or derived compounds classified as terpenes, alkaloids, polyketides, polyether phycotoxins, and quinazolinones were examined. In addition, one extract enriched in 13-acetoxysarcocrassolide and salmon oil enriched in omega-3 polyunsaturated fatty acids were also explored. These substances impacted cell viability across various in vitro and in vivo cancer models derived from diverse tissues, including the lung, prostate, breast, colorectal, stomach, skin, lymphatic system, kidney, and hematological malignancies such as leukemia and myeloma. These natural or derived marine compounds have been shown to target various biological biomarkers involved in cancer development and progression by activating distinct intracellular signaling pathways. These include mechanisms related to apoptosis, oxidative stress, mitochondrial dysfunction, and DNA fragmentation and condensation, as well as cell proliferation, cell migration, motility, invasion, and immune system activation.

In Contribution 1, Giani and co-workers critically review the biological activities of the rare C_50_ carotenoid called bacterioruberin (BR) and its derivatives, monoanhydrobacterioruberin and bisanhydrobacterioruberin. These compounds are produced by extreme halophilic microorganisms that inhabit hypersaline environments, where they are constantly exposed to ionic and oxidative stress due to saturated salt concentrations and high levels of UV radiation. These compounds demonstrated immunomodulatory, anti-inflammatory, and cytotoxic activities on various cellular models, and have been shown to modulate intracellular signaling pathways involved in key biological processes related to cancer, such as apoptosis and cell adhesion. The critical reflection conducted by Garcia and colleagues in Contribution 2 is focused on the potential of marine-derived compounds to disrupt hypoxic signaling pathways, inhibiting hypoxia-inducible factors (HIF-1 and HIF-2), given their critical roles in tumor metastasis and angiogenesis. Eighty-seven marine natural compounds were identified as being effective in modulating HIF activity. However, despite their potential, some of these compounds exhibit significant toxicity, and their mechanisms of action and validation in disease-relevant models remain limited. Therefore, further efforts are needed to optimize their efficacy, minimize toxicity, and improve pharmacokinetic profiles. In turn, in Contribution 3, Jiang and co-workers present a critical review focused on the discovery and development of novel variable new antigen receptors (VNARs), emphasizing shark VNARs as promising agents for the treatment and early diagnosis of tumors, particularly solid tumors. The VNAR from shark immunoglobulin novel antigen receptor (IgNAR) offers various advantages for anticancer development, including small size, high stability, high affinity, and distinct structural and functional characteristics. However, further research is needed to understand better the unique characteristics and therapeutic potential of IgNAR/VNAR in clinical and oncological applications.

The number of newly reported compounds of marine origin has grown over the last few decades. According to Faulkner, Blunt, and their collaborators, between 1977 and 2019, sponges (30.93%) were the leading source of new marine natural compounds, followed by microorganisms (20.53%) and seaweeds (10.44%) [4]. In 2023, more than 1200 new compounds were reported as having been isolated from marine microorganisms and phytoplankton; green, brown, and red algae; sponges; cnidarians; bryozoans; mollusks; tunicates; echinoderms; mangroves; and other intertidal plants. These molecules exhibited various activities, including cytotoxic and antiproliferative effects, as well as the ability to modulate intracellular signaling pathways relevant to therapeutic targets for the treatment of oncological diseases [5]. Herein, the compounds and marine-derived compounds studied were either isolated from or inspired by various marine sources, including algae, soft corals, fish, bacteria, and sponges. In Contribution 4, Lin and colleagues studied the anticancer potential of extracts from soft coral cultivated in aquaculture, focusing on their ability to interact with topoisomerase II, HDAC, and tubulin polymerization targets. Among the one hundred eighty ethyl acetate extracts tested, the *Lobophytum crassum* extract (LCE) displayed the smallest IC_50_ values on prostate cancer cells and significantly inhibited tubulin polymerization. Its effects on cell viability seemed to be mediated by apoptosis. In vivo, the extract was able to suppress tumor growth and reduce the tumor volume and weight. The LCE also displayed the ability to modulate various intracellular signaling pathways and biological events related to cell migration and invasion. Chemical analysis identified 13-AC (13-acetoxysarcocrassolide) as the major component of the LCE. In Contribution 5, Anh and co-workers explored the chemical diversity of compounds isolated from the culture broth of the marine-derived bacterium *Salinispora arenicola*, identifying five known (2–5 and 8) and three new (1, 6, and 7) rifamycin-related polyketide derivatives. After structural elucidation, the cytotoxic effects of these compounds were evaluated on several malignant cell lines. The rifamycin derivatives exhibited moderate to weak cytotoxicity, with evidence suggesting that the aromatic moiety plays an important role in their activity, particularly for the compound 1 that displayed the highest activity with GI_50_ values ranging from 2.36 to 9.96 µM.

In Contribution 6, Pulat and colleagues isolated a new quinazolinone derivative, actinoquinazolinone (**1**), from the culture of *Streptomyces* sp. CNQ-617, along with two known compounds. The new compound exhibited the strongest antitumor activity, demonstrating the ability to suppress invasion in AGS cells by modulating EMT and STAT3 signal pathways and the expression of various genes related to cell motility. In Contribution 7, Kim and co-workers investigated the anticancer effects of palytoxin, one of the most potent biotoxins, on various leukemia and solid tumor cell lines. The biotoxin isolated from the soft coral *Palythoa aff. Clavate* mediated selective cell death in leukemia cell lines, modulating the expression of several biological biomarkers related to apoptosis. In vivo, palytoxin inhibited the tumor formation on a zebrafish xenograft model at pM concentrations.

On the other hand, in Contribution 8, Tsai and colleagues evaluated the immunotherapeutic effects of crassolide, a cembranolide isolated from the Formosan soft coral *Lobophytum michaelae*, on in vitro/in vivo breast cancer models. This compound has been demonstrated to reduce the viability of human breast malignant cells and murine mammary carcinoma cells while also inducing immunogenic cell death (ICD) and decreasing the expression levels of CD24 on 4T1-luc2 cells. One of the major inducers of ICD is endoplasmic reticulum (ER) stress, in which the p38 MAPK signaling pathway plays a critical role. Crassolide upregulates the phosphorylation of p38α and downregulates the phosphorylation of NF-κB, STAT1, and EIK-1, key downstream effectors of the p38α signaling cascade. These findings suggest that crassolide may act as a novel p38 catalytic inhibitor.

Despite the potential of marine natural products as anticancer agents, the translation from laboratory findings to clinical trials has been limited, particularly due to low stability, bioavailability, water solubility, efficacy, pharmacokinetics, etc. [6]. Over the past few decades, various strategies have been developed to overcome these limitations, including the synthesis or hemi-synthesis of structurally related analogs. These modified molecules are designed to enhance the potency and efficacy of the original molecules as well as to mitigate their drawbacks. Accordingly, in Contribution 9, Alves and co-workers conducted the semi-synthesis of six novel sphaerococcenol A derivatives through reactions on the enone function group via thiol-Michael addition and enone reduction. These modifications did not contribute to potentiating the cytotoxic effects of the original compound. Of the analogs, compound (**1**) exhibited similar effects to those of sphaerococcenol A, accompanied by an increase in reactive oxygen species levels, changes in mitochondrial membrane potential, and the activation of apoptosis. In Contribution 10, Orfanoudaki and colleagues investigated the antitumor effects of twenty-four compounds from a library of naturally occurring and semi-synthetic discorhabdins on the viability of Merkel cell carcinoma cells. These compounds do not seem to induce apoptosis, but a rapid loss of cellular reducing potential and mitochondrial membrane potential suggests that they trigger mitochondrial dysfunction, leading to non-apoptotic cell death.

Chemoresistance, whether inherent or acquired, remains one of the major challenges in effective cancer treatment. It is often responsible for cancer recurrence, treatment failure, and, ultimately, patient death. Therefore, beyond the discovery and development of new molecules with anticancer activity and novel mechanisms of action, exploring their potential to enhance the efficacy of existing medicines is also relevant. Combination strategies may contribute to overcoming the limitations of single-agent therapies and contribute to more successful clinical outcomes [7]. In Contribution 11, Pettersen and co-workers explored the antitumor effects of salmon oil OmeGo (Hofseth BioCare), rich in fatty acids, including the n-3 PUFAs docosahexaenoic acid (DHA) and eicosahexaenoic acid (EPA), in colorectal malignant cell lines alone and in combination with the chemotherapeutic agent 5-fluorouracil (5-FU). OmeGo displayed to decrease the cell viability and potentiate the effects of 5-FU, suggesting that this strategy holds promise for further studies as an alternative treatment approach for patients with colorectal cancer.

Together, the eleven publications featured in this volume provide a compelling overview of the huge potential of marine natural products as scaffolds for developing next-generation therapeutic agents to fight cancer through various strategies. As Guest Editors, we sincerely acknowledge the valuable contributions of all researchers and reviewers involved in this Special Issue and the support from the editorial board and the *Marine Drugs* editorial office. Our special thanks go to Dr. Grace Qu for her dedicated assistance.
